# Assessment of the use of computed tomography colonography in early detection of peritoneal metastasis in patients with gastric cancer: A prospective cohort study

**DOI:** 10.1371/journal.pone.0261527

**Published:** 2022-01-25

**Authors:** Kenichi Iwasaki, Haruhiko Cho, Yukio Maezawa, Kazuhito Tsuchida, Kazuki Kano, Hirohito Fujikawa, Takanobu Yamada, Takashi Ogata, Takashi Oshima

**Affiliations:** 1 Department of Surgery, Tokyo Metropolitan Cancer and Infectious Diseases Center Komagome Hospital, Bunkyo, Tokyo, Japan; 2 Department of Gastrointestinal and Pediatric Surgery, Tokyo Medical University, Shinjuku, Tokyo, Japan; 3 Department of Gastrointestinal Surgery, Kanagawa Cancer Center, Yokohama, Kanagawa, Japan; University of Florida, UNITED STATES

## Abstract

Peritoneal metastasis (PM) is one of the most frequent forms of gastric cancer recurrence. In this study, we aimed to use computed tomography (CT) colonography (CTC) to detect signs of PM earlier in patients in whom PM was suspected but not yet diagnosed. CTC was used to evaluate patients with clinical symptoms or general CT findings that were suspicious but not sufficient to confirm PM. In total, 18 patients with suspected PM were enrolled. Ten patients (55.6%) had PM on CTC. Abnormal colonic deformities were identified at locations other than those of the lesions detected by general CT in seven patients. The sensitivity and specificity of CTC for the detection of PM were 83.3% and 100%, respectively. The median overall survival after CTC was 201 days in the CTC-positive group, which was significantly shorter than that in the CTC-negative group (945 days, p = 0.01). In the multivariate analysis, a positive CTC finding was the only factor independently associated with survival (p = 0.005). According to our experience with 18 patients, CTC can be an alternative to conventional imaging for early detection of PM. Further prospective studies with larger sample sizes are warranted to confirm and validate these findings.

**University hospital Medical Information Network Clinical Trials Registry (UMIN-CTR): Registration number**: UMIN000044167.

## Introduction

Gastric cancer is the fourth most frequently observed malignancy, with the third highest mortality rate worldwide [1]. Peritoneal metastasis (PM)/gastric cancer recurrence is associated with a poor prognosis and generally worsens the overall treatment outcomes while reducing the median survival time [2, 3]. PM is one of the most frequent forms of recurrence and is one of the poorest prognostic factors in patients with recurrent disease; it is observed in ~50% of patients with distant metastasis. Even if such patients can be treated, the expected survival is only ~9 months [4]. Probable reasons for the poor prognosis of PM include diagnostic difficulties and subsequent treatment delays. Thus, early detection of PM and prompt intervention may be crucial for improving survival outcomes [5].

Accurate diagnosis of PM is crucial for assessment and optional therapeutic methods; however, it is not always straightforward. Several noninvasive imaging methods have been used for PM detection in patients with gastric cancer, including endoscopic ultrasound (EUS), computed tomography (CT), and fluorodeoxyglucose positron emission tomography (FDG-PET); however, none of them have been completely accurate in diagnosing PM. In a meta-analysis of imaging methods used for diagnosing PM [6], the sensitivities of EUS, CT, and 18F-FDG PET were 0.34 (95% confidence interval [CI]: 0.10–0.69); 0.33 (95% CI: 0.16–0.56); and 0.28 (95% CI: 0.17–0.44), respectively. Additionally, Yoshikawa *et al*. [7] analyzed 123 initial-onset gastric cancer cases with PM in a preoperative setting, and the accuracy of CT in diagnosing PM was 37.4%. The low sensitivity of conventional CT in detecting PM has also been demonstrated in other reports [8, 9]. Although CT is the most advantageous modality due to its versatility and reproducibility, it cannot detect small PM, similar to EUS and PET. This low sensitivity could be attributed to the nature of PM, as it diffusely disseminates within the peritoneal cavity. Thus, it may be challenging to assess PM using CT images alone, and a more sensitive and objective imaging method is required.

CT colonography (CTC) is a diagnostic imaging method that was first reported in 1994 and is less invasive than conventional colonoscopy [10]. In addition to the detection of colorectal mucosal lesions, the detection of colon wall deformities via acquisition of three-dimensional (3D) air images of the whole colon is an advantage of this method [11]. In this study, we aimed to use CTC for early detection of PM in patients in whom PM was suspected based on clinical symptoms and general CT findings but was not yet diagnosed, and to administer anticancer agents in a timely and effective manner.

## Materials and methods

### Patients and methods

This prospective study was undertaken to assess the diagnostic feasibility of CTC for the detection of PM. CTC was performed to improve diagnostic performance in cases wherein PM/gastric cancer recurrence was suspected based on physical examination, laboratory tests, and imaging findings, but a definite diagnosis could not be established. CTC had not been previously used in our institute for diagnostic purposes, and had not been implemented as standard-of-care for a definitive diagnosis in patients with suspected PM. The study was approved by the Institutional Review Board of Kanagawa Cancer Center Hospital (KCC No.2010EPID-2) and was conducted in accordance with the principles of the Helsinki Declaration and the Japanese Ethical Guidelines for Clinical Studies. There was a possibility of failed PM detection by the CTC that could lead to treatment disadvantages, as this was the first prospective study to use CTC for early-stage diagnosis of PM. This was explained to every patient before the acquisition of written consent. All patients were assigned to this study for the purpose of research, and participation in the study was offered to all who met the inclusion criteria; each patient was given the right to reject participation. Patients who met the inclusion criteria but refused to join the research were provided standard-of-care follow-ups at the designated periods through physical examination, laboratory tests, and conventional imaging.

The inclusion criteria were: (i) histologically diagnosed gastric cancer via endoscopic biopsy or surgical specimen retrieval; (ii) suspected PM/recurrence of gastric cancer based on at least one of the following clinical findings: abnormal physical symptoms with causes that could not be explained by other diseases, elevation of serum tumor markers, and suspicious but not definitive signs of PM on conventional CT images; (iii) Eastern Cooperative Oncology Group (ECOG) performance status of 0, 1, or 2; (iv) oral intake ability; and (v) provision of written consent by each patient.

To minimize selection bias, patients with symptoms that severely affected their daily lives were excluded. Other exclusion criteria were: (i) inability to undergo bowel preparation; (ii) obvious intestinal stenosis; (iii) presence of massive ascites; and (iv) inability to undergo carbon dioxide (CO_2_) insufflation through the rectum.

The primary endpoint of this study was the diagnostic sensitivity of CTC for PM. The secondary endpoints included overall survival (OS) and progression-free survival (PFS). Ideally, a pathological diagnosis is required to confirm PM; however, suspicious PM lesions detected by CTC are difficult to confirm using endoscopy or laparoscopy. Thus, we adopted the wait-and-see method to confirm PM. Patients were followed-up until the definitive development of PM. In this cohort study, the treatment to be administered after PM diagnosis was not specified. Accordingly, decisions regarding the timing of treatment initiation and the treatments to be administered were made on a case-by-case basis after a discussion between the patient and attending doctors. However, fluorouracil-compound plus cisplatin was administered as first-line chemotherapy when the patient did not undergo gastrectomy or developed recurrence, with the interval between S-1 adjuvant chemotherapy and recurrence being <6 months. All oncological definitions were in accordance with the Japanese Classification of Gastric Carcinoma 15th edition [12].

### CTC procedure

Bowel preparation was performed by administering low-residue diets for the three meals on the day before CTC, along with oral administration of a laxative (50 g magnesium citrate diluted in 180 mL of water and 10 mL of 0.75% sodium picosulfate) at 3:00 PM on the same day. After the colon was inflated with sufficient CO_2_ through a rectal catheter with the patient in the left lateral decubitus position, CTC of the abdominal cavity was performed in the supine and side positions. The procedure was repeated in the prone position. All patients underwent the same process to ensure the reproducibility of CTC. Radiological interpretations regarding abnormal deformity and/or thickness of the colonic wall were made by the attending surgeons and radiologists.

### Definition of PM

Patients with at least one of the following findings on conventional CT were enrolled in this study: small amount of ascites, intestinal wall thickening, intestinal mesentery thickening, and intraperitoneal nodules. After CTC, a tentative diagnosis of PM was established by the attending doctors and radiologists based on abnormal deformity of the parts of the colonic wall without intraluminal tumors or fecal matter. A definitive diagnosis of PM was based on the appearance of massive ascites (ascites extending beyond the pelvic cavity to the abdominopelvic cavity), hydronephrosis, multiple enlarged intra-abdominal masses, and an overt peritoneal mass on conventional follow-up of CT images obtained within 6 (patients who underwent chemotherapy) or 3 (untreated patients) months after CTC.

### Statistical analyses

According to the results of previous reports [13–15], the diagnostic sensitivity of CTC has been estimated to be 60%, with a threshold value of 50% and an expected value of 75%. Using Simon’s two-stage method with a one-sided alpha value of 0.05 and a beta value of 0.7, we determined that a sample of 18 patients was required for this study. Interobserver variation between the attending doctors and radiologists was evaluated using kappa statistics. Kappa values of >0.81, 0.61–0.80, 0.41–0.60, and <0.41 were considered to represent almost perfect agreement, substantial agreement, moderate agreement, and slight agreement, respectively. The Kaplan–Meier method was used to determine the cumulative survival rate, and this was compared between the patients with positive and negative CTC findings using the log-rank test. OS was defined as the period between CTC and death. PFS was defined as the period between CTC and recurrence or death, whichever occurred earlier. All statistical analyses were performed using R for Windows (version 3.3.3; http://www.r-project.org/), and a p-value of <0.05 was considered statistically significant.

## Results

**Fig 1:**
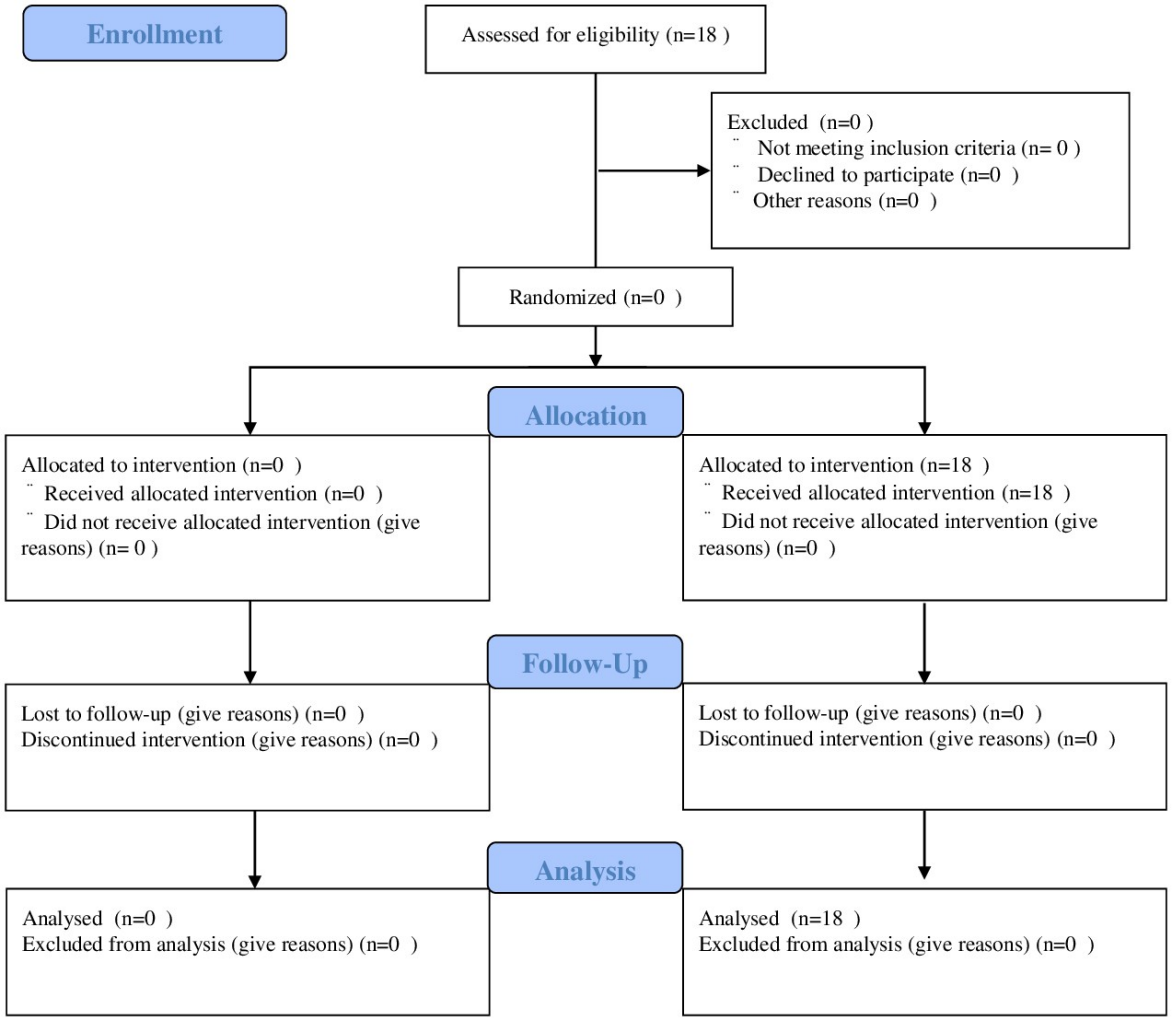
CONSORT 2010 flow diagram.

Since July 2010, CTC has been indicated for patients with suspected PM/recurrence of primary gastric carcinoma, as assessed by the attending surgeon at Kanagawa Cancer Center (Kanagawa, Japan). The final patient was registered in March 2016. As shown in Fig 1, Tables 1 and 2, CTC was performed in a total of 18 patients (11 men and 7 women; median age, 70 years). CTC was indicated for patients with clinical symptoms, abnormal serum tumor marker levels, and/or abnormal findings on conventional CT, which was the most common indication that was observed in 13 of the 18 patients. The treatments for PM were not regulated in this prospective cohort study.

**Table 1:** Patient characteristics.

	CTC negative (n = 8)	CTC positive (n = 10)
**Age, median (range)**	70 (54–88)	71.5 (57–83)
**Sex**		
Male	5	6
Female	3	4
**ECOG performance status**		
0/1	6/2	8/2
**Clinical criteria**		
**Clinical symptoms**		
Anorexia	3	1
Abdominal distension	0	3
Tenesmus	0	1
Nausea	0	2
Any	3	6
**TM elevation**		
CEA	0	4
CA 19–9	3	2
CA 125	1	2
Any	4	5
**Conventional CT findings**		
Small amount of ascites	1	3
Intestinal wall thickness	3	8
Intestinal mesentery thickness	1	0
Intraperitoneal nodules	0	0
Any	3	8

CTC, computed tomography colonography; ECOG, Eastern Cooperative Oncology Group; TM, tumor marker; CEA, carcinoembryonic antigen; CA, carbohydrate antigen.

**Table 2:** Primary stage and previous treatment for gastric cancer.

	CTC negative (n = 8)	CTC positive (n = 10)
**Type of gastrectomy**		
None	0	1
Distal	4	2
Total	4	7
**Extent of lymphadenectomy**		
<D2	1	1
≥D2	7	8
**Histological type**		
Differentiated	3	4
Undifferentiated	5	6
**pT**		
T1–T3	4	1
T4a, T4b	4	9
**pN**		
N0	1	1
N+	7	9
**Distant metastasis**		
M0	7	6
M1	1	4
**Pathological stage**		
II	2	0
III	5	6
IV	1	4
**Adjuvant chemotherapy**		
Yes	6	8
No	2	1

CTC, computed tomography colonography.

**Fig 2:**
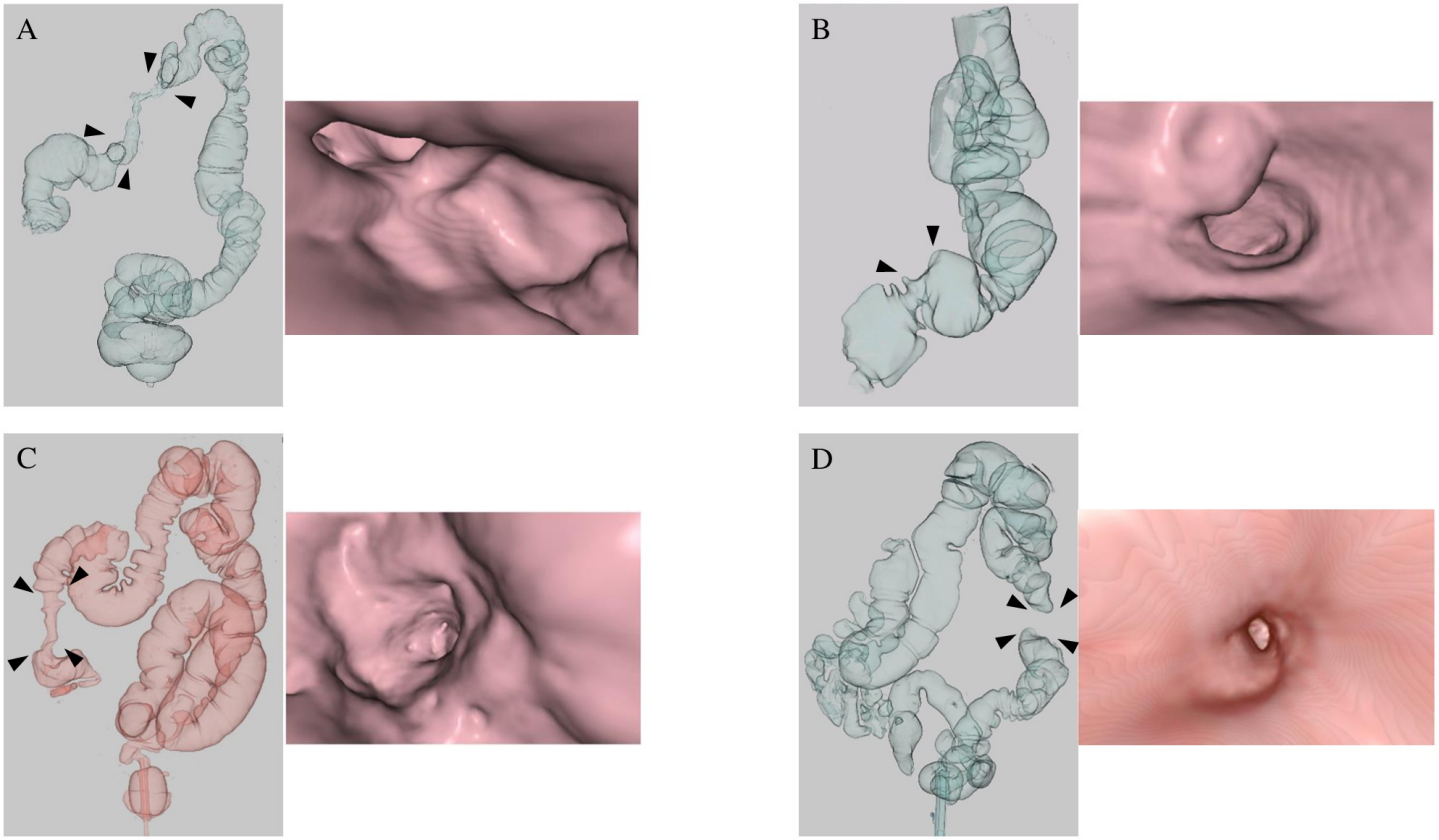
Representative computed tomography colonography (CTC) images.

Nine patients had clinical symptoms (anorexia, n = 4; abdominal distension, n = 3; nausea, n = 2), 10 had elevated tumor marker levels (carcinoembryonic antigen [CEA], n = 4; carbohydrate antigen (CA) 19–9, n = 5; CA125, n = 3), and 14 had suspicious findings suggestive of PM on axial CT images (thickening of the intestinal wall, n = 11; ascites, n = 4; intraperitoneal nodules, n = 0; thickening of the intestinal mesentery, n = 1). All four patients with a small amount of ascites on axial CT images also showed thickening of the intestinal wall and/or intestinal mesentery or intra-abdominal nodules. No CTC-related complications were observed. Representative positive CTC findings are shown in Fig 2. The surface-shaded reconstruction images and endoluminal images show a stricture in the transverse colon (A: black arrowhead), deformity of the sigmoid colon (B: black arrowhead), a stricture in the ascending colon (C: black arrowhead), and stenosis in the descending colon (D: black arrowhead). No neoplastic changes are observed on the mucosa surface in the endoluminal view.

**Fig 3:**
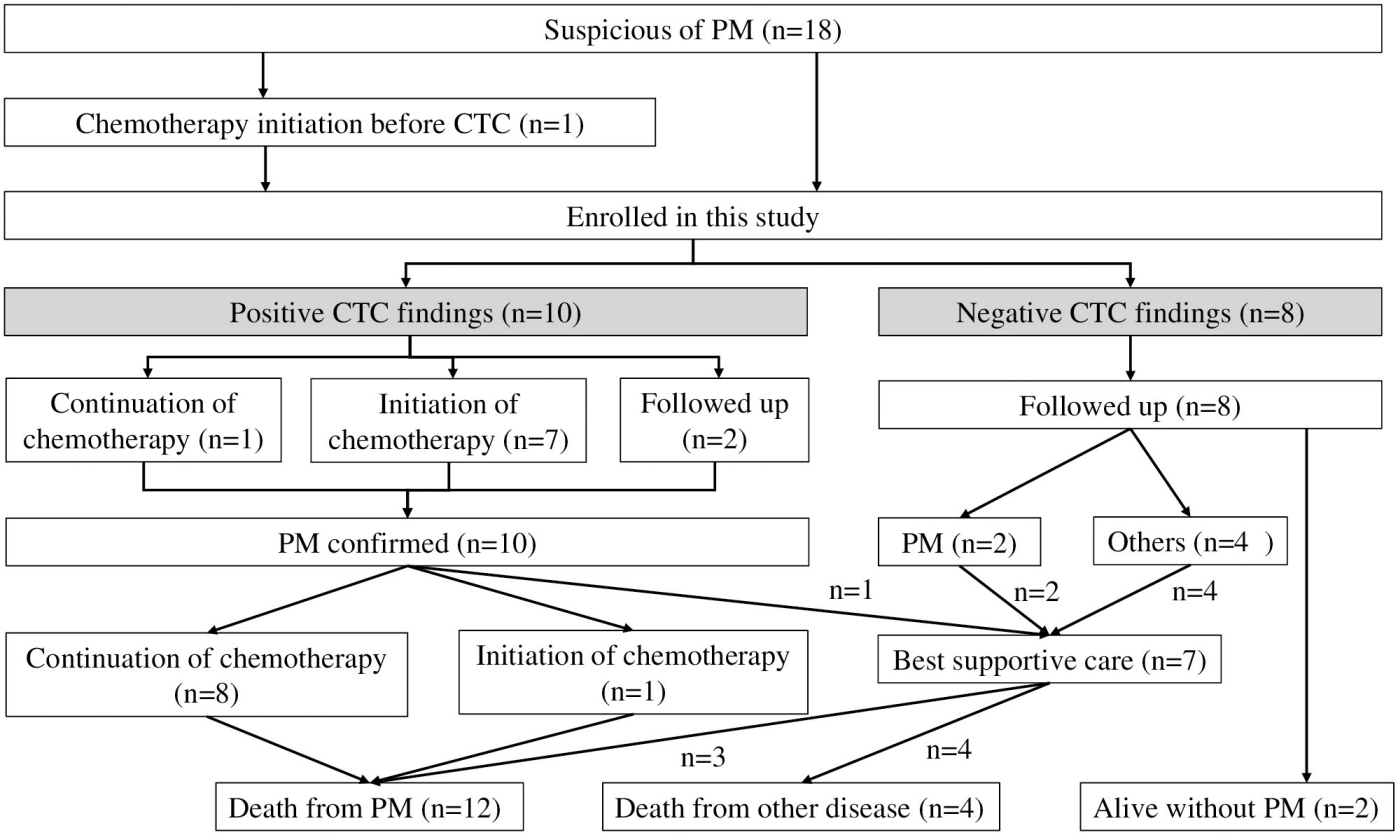
A flow diagram showing the treatment strategy for the 18 patients who underwent computed tomography colonography (CTC).

As shown in Fig 3, 10 patients (55.6%) showed positive findings that were suggestive of PM on CTC. Similarly, colonic deformities were identified at locations other than those of the lesions detected by general CT in seven patients. Treatment with anticancer agents was initiated in seven patients. One patient continued treatment, while two refused further treatment. The first-line chemotherapy regimens were as follows: S-1, n = 2; S-1 plus docetaxel, n = 2; paclitaxel, n = 2; and capecitabine plus cisplatin, n = 1. Second-line chemotherapy was administered when the patient experienced recurrence and the interval between S-1 adjuvant chemotherapy and recurrence was <6 months. Two patients received irinotecan, and one received capecitabine plus cisplatin as second-line chemotherapy. S-1 monotherapy was considered for patients who could feed orally, responded to S-1, and could not tolerate doublet chemotherapy due to old age or a poor general condition.

Six patients showed corresponding CTC and CT findings. In all cases with positive CTC findings, PM was clinically confirmed within 3 months. Among the eight patients who showed negative CTC findings, six (75%) did not develop recurrent PM, and two were alive without recurrence (Fig 2). The predictive accuracy, sensitivity, and specificity of conventional CT/CTC for the early detection of PM before obvious development were 61.1%/88.9%, 75%/83.3%, and 33.3%/100%, respectively (Table 3). The kappa value for correspondence between the attending surgeon and radiologist was 0.86, indicating an almost perfect agreement.

**Table 3:** Prediction of peritoneal metastasis (PM) development by conventional computed tomography (CT) and CT colonography (CTC).

	Conventional CT findings		CTC findings	
Definitive PM development	Positive	Negative	Positive	Negative
**Positive**	9	3	10	2
**Negative**	4	2	0	6

**Fig 4:**
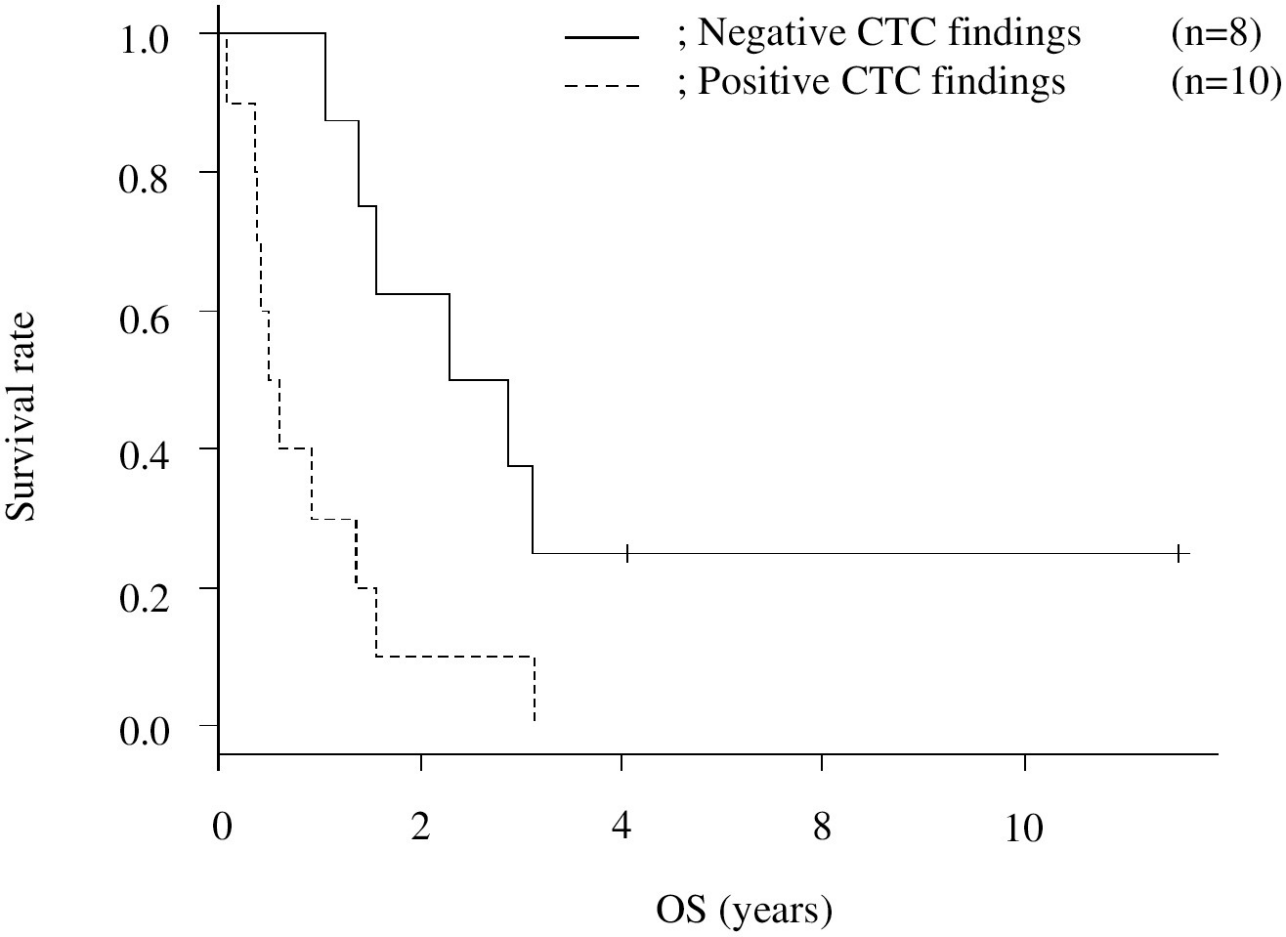
Kaplan–Meier survival curves for overall survival (OS) after computed tomography colonography (CTC).

**Fig 5:**
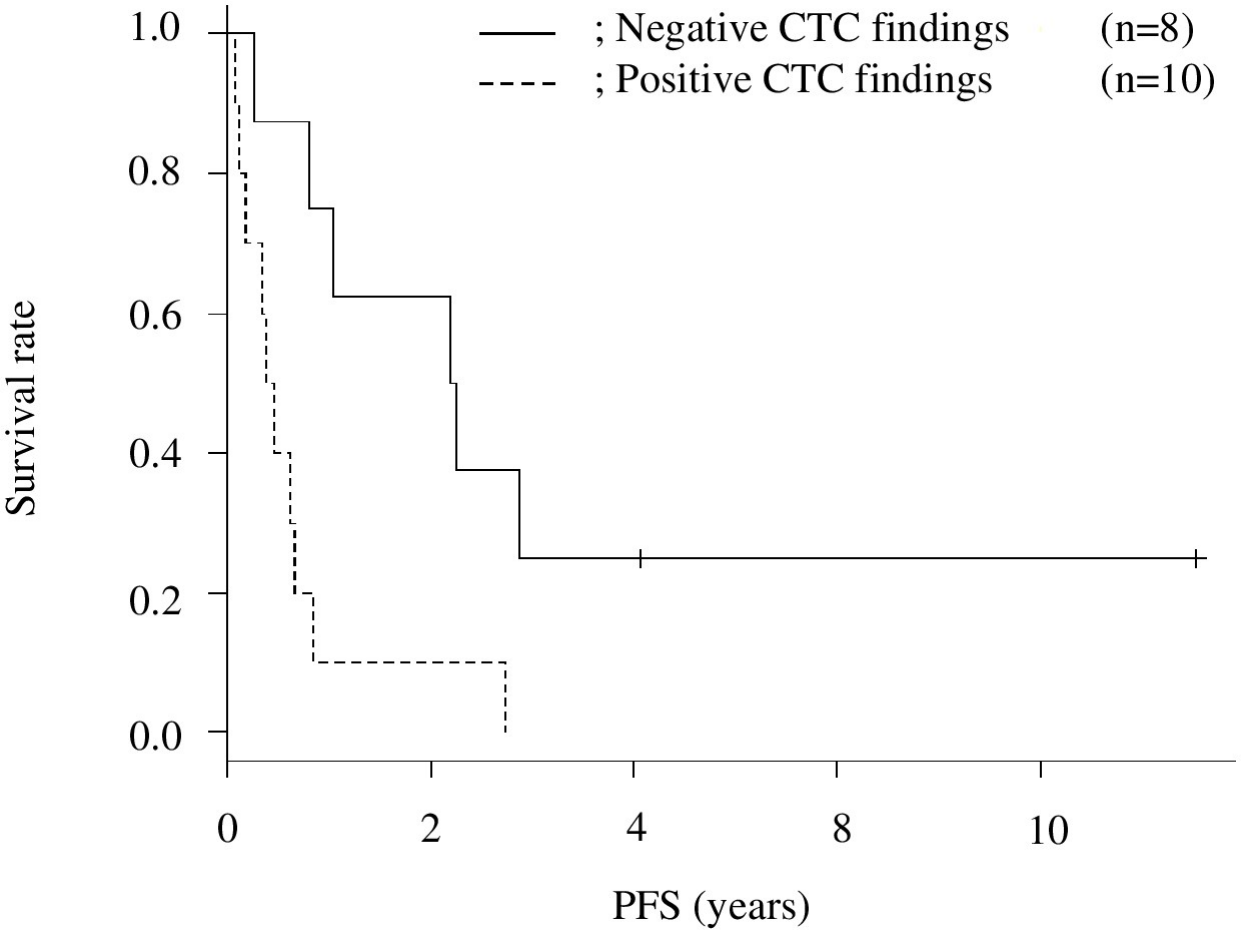
Kaplan–Meier survival curves for progression-free survival (PFS; clinical peritoneal metastasis-free period) after computed tomography colonography (CTC).

To address the prognostic role of positive CTC findings, we compared the OS between the CTC-positive and CTC-negative patients. The median OS periods in the CTC-positive and CTC-negative groups were 201 (95% CI: 29–495) days and 945 (95% CI: 386–NA) days (p = 0.01), respectively (Fig 4). Similarly, we collected the PFS data and compared them between the two groups, as shown in Fig 5. The median PFS periods in the CTC-positive and CTC-negative groups were 151 (95% CI: 28–238) and 810 (95% CI: 93–NA) days (p = 0.008), respectively. In the CTC-positive group, we compared the OS and PFS between the seven patients who started chemotherapy after CTC and the two patients who refused further treatment, including chemotherapy. The median OS periods in the chemotherapy and non-chemotherapy groups were 337 (95% CI: 141–709) days and 146 (95% CI: NA) days (p = 0.09), respectively; the median PFS periods were 226 (95% CI: 49–547) and 90 (95% CI: NA) days (p = 0.15), respectively. PM was the cause of death in all 12 patients with confirmed PM. Among these 12 patients, two had para-aortic lymph node metastasis. Previous reports have shown a poor prognosis of PM in patients without any treatment (3–6 months) and those with systematic chemotherapy (3.1–10.6 months) [4, 16]; our findings were consistent with those of previous studies.

As shown in Table 4, multivariate analysis of factors associated with OS revealed that a positive CTC finding was the only independent prognostic factor (p = 0.005).

**Table 4:** Univariate and multivariate Cox proportional hazards analyses of the clinicopathological factors associated with survival after CTC.

		Univariate analysis			Multivariate analysis		
Variables		HR	95% CI	P-value	HR	95% CI	P-value
Sex	Male	1.431	0.028–72.50	0.858			
	Female						
Age	<70 years	1.248	0.043–11.11	0.794			
	≥70 years						
PS	0	1.248	0.057–27.55	0.889			
	1						
pStage	II	2.768	0.098–77.80	0.550			
	III						
	IV						
Clinical symptoms	Positive	0.593	0.194–1.817	0.360			
	Negative						
TM elevation	Positive	0.599	0.027–12.90	0.743			
	Negative						
CT findings	Positive	0.409	0.118–1.414	0.158			
	Negative						
CTC findings	Positive	0.552	0.079–1.356	0.010	3.688	1.287–10.56	0.005
	Negative						
Adjuvant chemotherapy	Yes	0.260	0.045–1.497	0.131			
	No						

PS, performance status; TM, tumor marker; HR, hazard ratio; CI, confidence interval; CT, computed tomography; CTC, computed tomography colonoscopy.

## Discussion

To the best of our knowledge, this is the first prospective clinical study to explore the diagnostic and prognostic values of CTC for early detection of PM in gastric cancer. The diagnostic sensitivity of the CTC in our study was 83.3%, which was significantly higher than that of conventional CT (75%) and the statistically expected value (75%). Furthermore, CTC findings were associated with a worse prognosis.

CTC acquires 3D and virtual endoscopic images of the large intestine through computer data processing of CT images. It is less invasive and capable of detecting colorectal lesions with an accuracy that is comparable to that of colonoscopy, along with high objectivity and reproducibility. Thus, it offers a better method for PM visualization. We aimed to detect PM earlier by combining the expedient characteristics of both CT and contrast enema. In the 2011 National Comprehensive Cancer Network (NCCN) guidelines [17], CTC was recommended every 5 years with colonoscopy and every 10 years as a screening method for neoplastic polyps and cancer. Apart from providing an overview of the large intestine, any suspicious lesions appearing on 3D images can be visualized, which is an important advantage of this modality. Pickhardt *et al*. [18] have reported that cancers other than colorectal cancer could be identified at a rate of 1 in 300 cases. In our study, abnormal findings were observed in the same region of the large intestine on both axial CT and CTC images of seven patients. Interestingly, the CTC revealed additional findings in other regions in all patients. Moreover, no abnormal CTC findings were noted in four clinical PM-negative cases wherein PM was suspected on CT. These findings indicate that CTC is a feasible option for multifaceted evaluation of PM in patients with gastric cancer.

Elevation of the CA125 level reportedly correlates with the presence and expansion of PM in patients with gastric cancer and is an alternative marker for identifying PM. Takahashi *et al*. [19] have reported that the CA125-positive rate in patients with PM was high (42.9%), whereas the positive rates of other markers (CEA, CA19-9, and alpha fetoprotein) were 10% or lower; this result demonstrates high metastatic organ specificity. Wang *et al*. [20] have reported that the positive rate of CA125 measurement was 45.9%, in 269 PM-positive patients, while Nakata *et al*. [21] have reported a sensitivity and specificity of 39.4% and 95.7%, respectively. Although the sensitivity of CA125 measurement alone was observed to be only 46%, Nakata *et al*. have reported that a combination of multiple markers, such as CA72-4, increases the sensitivity [21]. In contrast, it has been frequently reported that the sensitivity of CA125 correlates with the degree of advancement of PM. According to Nakata *et al*., the accuracy of a CA125-based diagnosis was 28.6% when the lesion was at the P1 stage (vicinity of the stomach, small amount), whereas the diagnostic accuracy was 0% for both CT and US. Emoto *et al*. [16] have reported a strong correlation in the presence of ascites; however, the CA125 level exceeded the cutoff value in only one (12.5%) out of eight P1 cases. This suggests that CA125 cannot be expected to supplement the imaging diagnosis of PM in patients with gastric cancer. Another alternative method is contrast enema, which has long served as a useful tool for the detection of PM in Japan, considering that intestinal stenosis occurs frequently as PM advances in numerous patients with gastric cancer. However, contrast enema is less reliable due to the lack of reproducibility; furthermore, unlike CT, it cannot detect ascites or extraintestinal masses. If CT images are acquired while air is injected without contrast enema into the large intestine, the sensitivity of CT in detecting PM can be expected to improve.

PM in gastric cancer impairs the patient’s quality of life by obstructing the digestive tract or by frequently causing ascites that results in a poor prognosis [22, 23]. Ideally, early detection of PM by less invasive and clinically feasible CTC may lead to appropriate initiation of chemotherapy and, consequently, improved survival. In our study, the median OS period in the CTC-positive group was 201 days, which is far from satisfactory. However, when compared with the poor induction rate of 25% in the group of patients who started chemotherapy after PM was confirmed, all patients in the CTC-positive group could have started chemotherapy without delay. However, early detection and induction of treatment for PM did not prolong the OS or PFS in our study. This may be related to the very limited response of PM to systemic chemotherapy. Consistent with our results, the results of previous studies have reported a slim chance of achieving cure, with a 1-year OS rate and median OS period of 16%–40.7% and 3.1–10.6 months, respectively [4, 24, 25]. In contrast, CTC showed extremely high specificity, and the median OS period for patients without abnormal findings in CTC was 31.5 months. These results suggest that positive CTC findings can be associated with a poor prognosis in gastric cancer.

This study has several limitations. First, the sample size estimation was based only on sensitivity, and specificity was not considered at the beginning of this study since conventional modalities had been reported to have high specificity. We re-calculated the sample size required for this study using the area under the curve (AUC) of the c-statistics for prediction of definitive PM. The AUC was 0.542 when conventional CT was used to predict definitive PM. When we set the AUC with a threshold value of 0.6 and expected value of 0.85 for predicting PM by CTC, the required sample size was estimated to be 16 patients with a one-sided alpha of 0.05 and a power of 0.7. Actual AUC of CTC was 0.917, which was higher than the expected value. Second, the sample size of 18 patients was relatively small and could have led to bias; therefore, a larger number of cases is required to draw stronger conclusions. Third, the regimen and timing of the initiation of chemotherapy were not standardized, which may have caused variations in the study results. A study design constructed to include the chemotherapy regimen based on the possibility of oral intake and the function of other organs during treatment may overcome this limitation. Fourth, PM was not pathologically confirmed. Finally, only axial CT images were used for the analysis; coronal or sagittal views would have been useful for detecting PM in gastric cancer in some cases. This may have influenced our results and would need to be resolved in future studies.

## Conclusions

According to our experience with 18 patients, CTC may be an alternative to conventional imaging in detecting PM early in patients with gastric cancer. Further prospective studies with larger sample sizes are warranted to confirm and validate these findings.

## Supporting information

S1 Protocol (DOCX)

S2 Protocol (DOCX)

S1 Dataset (XLSX)

S1 Checklist (DOC)

## References

[1] Machlowska J, Baj J, Sitarz M, Maciejewski R, Sitarz R. Gastric cancer: epidemiology, risk factors, classification, genomic characteristics and treatment strategies. Int J Mol Sci. 2020;21(11):E4012. doi: 10.3390/ijms21114012 32512697 PMC7312039

[2] Koizumi W, Narahara H, Hara T, Takagane A, Akiya T, Takagi M, et al. S-1 plus cisplatin versus S-1 alone for first-line treatment of advanced gastric cancer (SPIRITS trial): a phase III trial. Lancet Oncol. 2008;9(3):215–221. doi: 10.1016/S1470-2045(08)70035-4 18282805

[3] Thomassen I, van Gestel YR, van Ramshorst B, Luyer MD, Bosscha K, Nienhuijs SW, et al. Peritoneal carcinomatosis of gastric origin: a population-based study on incidence, survival and risk factors. Int J Cancer. 2014;134(3):622–628. doi: 10.1002/ijc.28373 23832847

[4] Shirao K, Boku N, Yamada Y, Yamaguchi K, Doi T, Goto M, et al. Randomized Phase III study of 5-fluorouracil continuous infusion vs. sequential methotrexate and 5-fluorouracil therapy in far advanced gastric cancer with peritoneal metastasis (JCOG0106). Jpn J Clin Oncol. 2013;43(10):972–980. doi: 10.1093/jjco/hyt114 24014884

[5] Hasegawa H, Fujitani K, Nakazuru S, Hirao M, Yamamoto K, Mita E, et al. Optimal treatment change criteria for advanced gastric cancer with non-measurable peritoneal metastasis: symptom/tumor marker-based versus CT-based. Anticancer Res. 2014;34(9):5169–5174. 25202110

[6] Wang Z, Chen JQ. Imaging in assessing hepatic and peritoneal metastases of gastric cancer: a systematic review. BMC Gastroenterol. 2011;11:19. doi: 10.1186/1471-230X-11-19 21385469 PMC3062583

[7] Yoshikawa T, Kanari M, Tsuburaya A, Kobayashi O, Sairenji M, Motohashi H. [Clinical and diagnostic significance of abdominal CT for peritoneal metastases in patients with primary gastric cancer]. Gan To Kagaku Ryoho. 2002;29(11):1925–1928. Japanese. 12465391

[8] Kim SJ, Kim HH, Kim YH, Hwang SH, Lee HS, Park DJ, et al. Peritoneal metastasis: detection with 16- or 64-detector row CT in patients undergoing surgery for gastric cancer. Radiology. 2009;253(2):407–415. doi: 10.1148/radiol.2532082272 19789243

[9] Davies J, Chalmers AG, Sue-Ling HM, May J, Miller GV, Martin IG, et al. Spiral computed tomography and operative staging of gastric carcinoma: a comparison with histopathological staging. Gut. 1997;41(3):314–319. doi: 10.1136/gut.41.3.314 9378384 PMC1891482

[10] Vining DJ, Gelfand DW, Bechtold RE, Scharling ES, Grishaw EK, Shifrin RY. Technical feasibility of colon imaging with helical CT and virtual reality. AJR Am J Roentgenol. 1994;162:S104.

[11] Tomimatsu H, Kanematsu M, Goshima S, Watanabe H, Ono H, Asano T, et al. Uneven haustra on CT colonography: a clue for the detection of transperitoneal invasion from gastric cancer. Abdom Imaging. 2012;37(4):570–574. doi: 10.1007/s00261-011-9819-5 22038331

[12] Japanese Gastric Cancer Association. Japanese Classification of Gastric Carcinoma. 15th ed. Tokyo: Kanehara; 2017. 10.1007/s101209800016 11957040

[13] Whiting J, Sano T, Saka M, Fukagawa T, Katai H, Sasako M. Follow-up of gastric cancer: a review. Gastric Cancer. 2006;9(2):74–81. doi: 10.1007/s10120-006-0360-0 16767361

[14] Kwee RM, Kwee TC. Imaging in local staging of gastric cancer: a systematic review. J Clin Oncol. 2007;25(15):2107–2116. doi: 10.1200/JCO.2006.09.5224 17513817

[15] Chan DY, Syn NL, Yap R, Phua JN, Soh TI, Chee CE, et al. Conversion surgery post-intraperitoneal paclitaxel and systemic chemotherapy for gastric cancer carcinomatosis peritonei. are we ready. J Gastrointest Surg. 2017;21(3):425–433. doi: 10.1007/s11605-016-3336-3 27981493

[16] Emoto S, Ishigami H, Yamashita H, Yamaguchi H, Kaisaki S, Kitayama J. Clinical significance of CA125 and CA72-4 in gastric cancer with peritoneal dissemination. Gastric Cancer. 2012;15(2):154–161. doi: 10.1007/s10120-011-0091-8 21892754

[17] nccn.org [Internet]. National Comprehensive Cancer Network (NCCN) Clinical Practice Guidelines in Oncology: Colorectal Cancer Screening. c(year) [cited (insert date here)] http://www.nccn.org/. 10.6004/jnccn.2010.0003 20064289

[18] Pickhardt PJ, Kim DH, Meiners RJ, Wyatt KS, Hanson ME, Barlow DS, et al. Colorectal and extracolonic cancers detected at screening CT colonography in 10,286 asymptomatic adults. Radiology. 2010;255(1):83–88. doi: 10.1148/radiol.09090939 20308446

[19] Takahashi Y. [Gastrointestinal cancer]. Gan To Kagaku Ryoho. 2004;31(8):1275–1279. Japanese. 15332558

[20] Wang Q, Yang Y, Zhang YP, Zou Z, Qian X, Liu B, et al. A Prognostic value of carbohydrate tumor markers and inflammation-based markers in metastatic or recurrent gastric cancer. Med Oncol. 2014;31(12):289. J Jpn Surg Assoc. 1994;55(8):1932–1937. doi: 10.1007/s12032-014-0289-9 25344872

[21] Nakata B, Hirakawa-YS Chung K, Kato Y, Yamashita Y, Maeda K, Onoda N, et al. Serum CA 125 level as a predictor of peritoneal dissemination in patients with gastric carcinoma. Cancer. 1998;83(12):2488–2492. 9874453 10.1002/(sici)1097-0142(19981215)83:12<2488::aid-cncr12>3.0.co;2-1

[22] Japanese Gastric Cancer Association Registration Committee; Maruyama K, Kaminishi M, Hayashi K, Isobe Y, Honda I, Katai H, et al. Gastric cancer treated in 1991 in Japan: data analysis of nationwide registry. Gastric Cancer. 2006;9(2):51–66. doi: 10.1007/s10120-006-0370-y 16767357

[23] Maehara Y, Kakeji Y, Oda S, Takahashi I, Akazawa K, Sugimachi K. Time trends of surgical treatment and the prognosis for Japanese patients with gastric cancer. Br J Cancer. 2000;83(8):986–991. doi: 10.1054/bjoc.2000.1427 10993643 PMC2363551

[24] Sadeghi B, Arvieux C, Glehen O, Beaujard AC, Rivoire M, Baulieux J, et al. Peritoneal carcinomatosis from non-gynecologic malignancies: results of the EVOCAPE 1 multicentric prospective study. Cancer. 2000;88(2):358–363. 10640968 10.1002/(sici)1097-0142(20000115)88:2<358::aid-cncr16>3.0.co;2-o

[25] Ohtsu A, Shimada Y, Shirao K, Boku N, Hyodo I, Saito H, et al. Randomized phase III trial of fluorouracil alone versus fluorouracil plus cisplatin versus uracil and tegafur plus mitomycin in patients with unresectable, advanced gastric cancer: The Japan Clinical Oncology Group Study (JCOG9205). J Clin Oncol. 2003;21(1):54–59. doi: 10.1200/JCO.2003.04.130 12506170

