# Supplementary material for: Assessment of the use of computed tomography colonography in early detection of peritoneal metastasis in patients with gastric cancer: A prospective cohort study
Source: PLoS One. 2022 Jan 25;17(1):e0261527. doi: 10.1371/journal.pone.0261527 (PMC8789127; doi:10.1371/journal.pone.0261527)
Supplement: S1 Protocol — (DOCX) [file pone.0261527.s001.docx]

**Feasibility study of CT colonography for peritoneal metastasis of gastric cancer clinically difficult to diagnose definitely**

**Principal Investigator** Haruhiko Cho

Department of Gastrointestinal Surgery, Kanagawa Cancer Center 2-3-2 Nakao, Asahi Ward, Yokohama, Kanagawa 241-851, Japan

Tel.: +81- 45-520-2222, Fax : +81-45-361-4692

E-mail: choharuhiko@kcch.jp

**Study Secretariat** Tetsuo Yoshida

Department of Radiology, Kanagawa Cancer Center 2 -3-2 Nakao, Asahi Ward, Yokohama, Kanagawa 241-851, Japan

Tel.: +81- 45-520-2222, Fax : +81-45-361-4692

E-mail: jsbach@kcch.jp

Akira Tuburaya

Department of Gastrointestinal Surgery, Kanagawa Cancer Center 2-3-2 Nakao, Asahi Ward, Yokohama, Kanagawa 241-851, Japan

Tel.: +81- 45-520-2222, Fax : +81-45-361-4692

E-mail: tuburayaa@kcch.jp

Ver1.0 2010/9/20

1. Outline

0.1. Schema

Advanced Gastric cancer or

postoperative cases suspicious for recurrence

・Suspicious of PM: 1 to 3 of following three clinical criteria

1) Symptoms

Pain, Abdominal distention, Nausea, Tenesmus

2) Tumor marker

Elevation of CEA and/or CA19-9 and/or CA125

3) Images

Ascites, Thickness of intestinal wall and/or intestinal mesentery.

・ECOG performance status : 0/1/2

・ No bowel obstruction

・ Written Informed consent

CT colonography

0.2. Aim

CTC will be performed aiming at improvement of diagnostic performance in patients in whom peritoneal metastasis/recurrence of stomach cancer is suspected based on physical, laboratory test, and imaging findings, but no definite diagnosis can be made. The presence or absence of deformation suggesting peritoneal dissemination in the large intestinal wall will be diagnosed jointly by the physician in charge and radiologist and correlation with the clinical course will be analyzed.

0.3. Subjects

1. Histologically diagnosed with stomach cancer by endoscopic biopsy or surgical biopsy.
2. Peritoneal metastasis/recurrence of stomach cancer is suspected based on physical, laboratory test, and imaging findings.
3. The ECOG performance status is 0, 1, or 2.
4. Oral ingestion is possible.
5. Written consent is obtained from the patient.

0.4.Target number of enrollment

The diagnostic sensitivity of CTC has been estimated to be 60%, with a threshold value of 50% and an expected value of 75%. Using Simon’s two-stage method with a one-sided alpha value of 0.05 and a beta value of 0.7, we determined that a sample of 18 patients was required for this study.

1.Aim

In this study, we aimed to use CTC for early detection of PM in patients in whom PM was suspected based on clinical symptoms and general CT findings but not yet diagnosed, and to administer anticancer agents in a timely and effective manner.

2.Background and bases of plan

2.1. Object

2.1.1. Object disease

The mortality from stomach cancer is the second highest following that from lung cancer in Japan. It is considered that highly advanced/recurrent stomach cancer reduces the overall treatment outcome and MST of the S-1 group in these patients was 13.0 month in the SPIRITS study 1). Peritoneal metastasis/recurrence was the most frequent as well as being the poorest prognostic recurrence pattern accounting for about half of patients with distant metastasis, and even if they can be treated, only about 9-month survival can be expected (JCOG0106). The cause of poor prognosis of peritoneal metastasis includes the following conditions other than malignancy of cells: A sufficient amount of anticancer drugs cannot be administered because intestinal stenosis and hydronephrosis are likely to occur. Identification by imaging is difficult and initiation of treatment is delayed. The disease may spread to every region in the abdominal cavity and it is difficult to get the entire picture. Since evaluation of treatment is difficult, treatment cannot be rapidly switched when the disease is resistant.

2.2. Diagnoses

2.2.1. Conventional imaging methods

The imaging diagnosis rate of peritoneal dissemination of stomach cancer is low and the odds ratio of CT for diagnosing peritoneal dissemination was 66.18 (95%CI: 27.28-160.53), being the highest in a meta-analysis of 33 reports (US: 8, EUS: 5, CT: 22, MRI: 2, FDG-PET: 5) 2). In all diagnostic methods, the diagnostic specificity was 0.96-0.99, being high, but even the diagnostic sensitivity of EUS with the highest sensitivity was only 0.34 (0.10-0.69) and that of CT was 0.33 (0.16-0.56), showing that peritoneal recurrence may be overlooked on axial CT normally employed for postoperative follow-up of stomach cancer in 2 out of 3 cases. In a randomized controlled study performed in Korea 3), CT and FDG-PET were compared. Intra-abdominal nodules, ascites, irregularity with beaded thickening of the mesentery and omentum or fluffy images were regarded as peritoneal dissemination on CT. On PET, diffuse metabolic enhancement along the intestine and mesentery was set as a judgment criterion. The sensitivity of CT was 76.5%, being significantly higher than that (35.3%) of PET (p=0.037), but the specificity was 91.6%, being inferior to that (98.9%) of PET (p=0.035). Yoshikawa et al. 4) analyzed 123 initial-onset stomach cancer cases with peritoneal metastasis and observed that the accuracy of diagnosing peritoneal dissemination as positive on preoperative diagnosis by CT was 37.4%, being low, but the outcome was significantly poorer in the positive than negative group, showing its significance as a prognostic factor.

2.2.2. Tumor markers

Regarding serum tumor marker, elevation of the CA125 level in stomach cancer patients is correlated with the presence and expansion of peritoneal dissemination. Takahashi 5) reported that the CA125-positive rate in patients with peritoneal dissemination of stomach cancer was high (42.9%) whereas those of other markers (CEA, CA19-9, and AFP) are 10% or lower, showing high metastatic organ specificity. The reason is considered as follows: CA125 is not produced by tumor cells, but it is present in peritoneal mesothelial cells, so that it represents peritoneal inflammation. Accordingly, infectious peritonitis, endometriosis of females, and early period after surgery (the half-life of CA125 is 5-10 days 6)) have to be excluded. Ueki et al. 7) measured CA125 in 219 patients with surgical stomach cancer before surgery and observed that the sensitivity and specificity of CA125 were 31.9 and 93.6%, respectively, in 47 peritoneal dissemination-positive patients. Nakata et al. 8) reported that the sensitivity and specificity of CA125 for peritoneal dissemination of stomach cancer were 39.4 and 95.7%, respectively, showing its superiority to imaging diagnosis including CT and US. Emoto et al. 9) reported that combination with multiple markers, such as CA72-4, increases the sensitivity, but the sensitivity of CA125 alone was only 46%.

On the other hand, it has been frequently reported that the sensitivity of CA125 is correlated with the degree of advancement of peritoneal dissemination, so that it does not become positive when the absolute amount of tumor is still small. According to Nakata et al., the accuracy of CA125-based diagnosis was 28.6% when the stage was P1 (vicinity of the stomach, small amount), and those of CT and US were 0%. In a study reported by Emoto et al., correlation was high when ascites was present, but the CA125 level exceeded the cutoff value in only 1 (12.5%) of 8 P1 cases, suggesting that CA125 cannot be expected to supplement imaging diagnosis of peritoneal dissemination of stomach cancer.

2.2.3. Contrast enema

Since intestinal stenosis occurs as peritoneal dissemination advances in many cases, when stenosis is observed in stomach cancer patients on contrast enema, peritoneal dissemination is strongly suspected. However, the diagnostic sensitivity of contrast enema is unclear when it is performed while the absolute amount of disseminated tumor is still small, even though characteristic findings, such as “striped colon 10)” and “fixed transverse parallel folds 11)”, are observed. Miyagawa 12) reported comparisons between contrast enema and CT findings in 60 patients diagnosed with large intestinal metastasis of stomach cancer. The definite diagnosis of metastasis was made by surgery in 28, autopsy in 5, and clinical judgment, such as imaging, in 27. When 5 mm or more wall thickness on CT was set as a diagnostic criterion, the diagnosis could be made in more than 80%, but the diagnostic sensitivity of enema was 98% (59/60), being superior to that (83% (50/60)) of CT. On the other hand, CT was considered advantageous when the purpose was diagnosis of peritoneal dissemination, because intra-abdominal masses were noted in 62% of the patients and ascites was noted in 37%, showing that the lesions are derived from peritoneal dissemination in many patients. One reason for the absence of overlooking on enema is that the difference between normal and metastatic regions is emphasized by dilating the large intestine by injecting air. If CT images are acquired while air is injected into the large intestine, improvement of the sensitivity of CT for peritoneal dissemination can be expected.

2.2.4. CT colonography

CT colonography (CTC) is an imaging diagnosis method initially reported in 1994. It acquires 3D and virtual endoscopic images of the large intestine by computer data processing of CT images. It is low-invasive and capable of detecting colorectal lesions at an accuracy comparable to that of colonoscopy. In the 2011 NCCN guidelines 13), every 5-year CTC is stated with every 10-year colonoscopy as a screening method to discover neoplastic polyps and cancer. In addition, information other than that of the large intestine can be acquired and this is also a big advantage. Pickhardt et al. 14) reported that cancers other than colorectal cancer were identified at a rate of one in 300 cases. However, to our knowledge, no study performing CTC to detect minute extramural changes in the large intestine has been reported as of January 2020.

Advantages of diagnostic images of CTC include high objectivity and reproducibility, being a technique appropriate for standardization. In Japan, imaging of the lower digestive tract by CTC has been covered by national health insurance since January 2012, and 600 medical fee points are added when carbon dioxide is injected using a tube for the rectum and 3-dimensional imaging process is performed using 16- or more row multi-slice CT.

2.3. Study design

2.3.1. Endpoint

The primary endpoint of this study was the diagnostic sensitivity of CTC for PM. The secondary endpoints included overall survival (OS) and progression-free survival (PFS). The diagnostic sensitivity of CTC has been estimated to be 60%, with a threshold value of 50% and an expected value of 75%. Using Simon’s two-stage method with a one-sided alpha value of 0.05 and a beta value of 0.7, we determined that a sample of 18 patients was required for this study.

2.3.2. Patient enrollment expectancy

The recurrence rate of post surgical gastric cancer patients is 10-20%. That will calculate approximately 30 patients a year. Among those cases, five to eight will be predicted to show PM.

3. Patient selection

The patients were cases wherein PM/gastric cancer recurrence was suspected based on physical examination, laboratory tests, and imaging findings, but a definite diagnosis could not be established.

3.1. Inclusion criteria

(i) histologically diagnosed gastric cancer via endoscopic biopsy or surgical specimen retrieval; (ii) suspected PM/recurrence of gastric cancer based on at least one of the following clinical findings: abnormal physical symptoms with causes that could not be explained by other diseases, elevation of serum tumor markers, and suspicious but not definitive signs of PM on conventional CT images; (iii) Eastern Cooperative Oncology Group (ECOG) performance status of 0, 1, or 2; (iv) oral intake ability; and (v) provision of written consent by each patient.

3.2. Exclusion criteria were

(i) inability to undergo bowel preparation; (ii) obvious intestinal stenosis; (iii) presence of massive ascites; and (iv) inability to undergo carbon dioxide (CO2) insufflation through the rectum.

4. Registration

4.1. Registration procedure

Check the inclusion and exclusion criteria and contact below.

Data center: Rika Takahashi

Department of Gastrointestinal Surgery, Kanagawa Cancer Center

Tel.: +81- 45-391-5761 (internal 2633)

Fax : +81-45-361-4692

5.Procedure

5.1. Standardized procedure

5.1.1. Preparation

Bowel preparation was performed by administering low-residue diets for the three meals on the day before CTC, along with oral administration of a laxative (50 g magnesium citrate diluted in 180 mL of water and 10 mL of 0.75% sodium picosulfate) at 3:00 PM on the same day.

5.1.2 CTC

1.After the colon was inflated with sufficient CO2 through a rectal catheter with the patient in the left lateral decubitus position.

2.CTC of the abdominal cavity was performed in the supine and side positions. The procedure was repeated in the prone position.

3.All patients underwent the same process to ensure the reproducibility of CTC.

5.2. Interpretation

Radiological interpretations regarding abnormal deformity and/or thickness of the colonic wall were made by the attending surgeons and radiologists.

6. Predicted adverse events

Along with the procedure of CTC, adverse events such as gastrointestinal perforation, gastrointestinal bleeding and bleeding will be dealt appropriately whenever occurred.

7. Ethical matters

All researchers involved in this study act in conformity to the principles of the Helsinki Declaration (http://www.med.or.jp/wma/) and the Japanese Ethical Guidelines for Clinical Studies(http://www.mhlw.go.jp/topics/2003/07/tp0730-2.html).

7.1. Explanation and acquisition of written consent

The attending surgeon provided the oral explanation by using the document approved by the ethic screening committee (or Institutional Review Board，IRB) before registering, and obtain an agreement by the free will of the person with written consent. When a study cooperator gives a supplementary explanation other than the medical attendant whom explained, a signature seals it after filling out each date or the study cooperator concerned signs written consent and a signature seals the person of object after filling out an agreement day or signs it. The copy of written consent will be provided to the patient and the original will be kept in facilities.

7.2. Privacy and patient identification

When handling the data and consent forms related to the implementation of the research, sufficient consideration will be given to protecting the confidentiality of the subjects. In addition, when preparing reports to be submitted outside the hospital, the subjects will be identified by the "case registration number" issued at the time of registration allocation and the "subject identification code" set within the facility, and confidentiality will be protected. The data obtained in this study will be used only for the purpose of this study.

7.3. Approval by the Institutional Review Board

Prior to the initiation of this study, each institution must submit the required documentation and be approved for participation in the study by an expedited review of the institution's IRB or other means.

7.4. Conflict of interest

There is no conflict of interest related to this study.

8.Research facility

8.1. Principal Investigator

Haruhiko Cho

Department of Gastrointestinal Surgery, Kanagawa Cancer Center 2-3-2 Nakao, Asahi Ward, Yokohama, Kanagawa 241-851, Japan

Tel.: +81- 45-520-2222, Fax : +81-45-361-4692

E-mail: choharuhiko@kcch.jp

8.2. Study Secretariat

Tetsuo Yoshida

Department of Radiology, Kanagawa Cancer Center 2 -3-2 Nakao, Asahi Ward, Yokohama, Kanagawa 241-851, Japan

Tel.: +81- 45-520-2222, Fax : +81-45-361-4692

E-mail: jsbach@kcch.jp

Akira Tuburaya

Department of Gastrointestinal Surgery, Kanagawa Cancer Center 2-3-2 Nakao, Asahi Ward, Yokohama, Kanagawa 241-851, Japan

Tel.: +81- 45-520-2222, Fax : +81-45-361-4692

E-mail: tuburayaa@kcch.jp

8.3. Data center

Rika Takahashi

Department of Gastrointestinal Surgery, Kanagawa Cancer Center

Tel.: +81- 45-391-5761 (internal 2633)

Fax : +81-45-361-4692

8.4. Research project document execute committee

Haruhiko Cho: Department of Gastrointestinal Surgery, Kanagawa Cancer Center

Takaki Yoshikawa: Department of Gastrointestinal Surgery, Kanagawa Cancer Center

Tetsuo Yoshida: Department of Radiology, Kanagawa Cancer Center

9. References

1) Koizumi W, et al. S-1 plus cisplatin versus S-1 alone for first-line treatment of advanced gastric cancer (SPIRITS trial): a phase III trial. Lancet Oncol 9: 215-221, 2008.

2) Wang Z, Chen JQ. Imaging in assessing hepatic and peritoneal metastases of gastric cancer: a systematic review. BMC Gastroenterol 11: 19, 2011.

3) Kim DW, Park SA, Kim CG. Detecting the recurrence of gastric cancer after curative resection: comparison of FDGPET/CT and contrast-enhanced abdominal CT. J Korean Med Sci 26: 875-880, 2011.

4) Yoshikawa T, Kanari M, Tsuburaya A, Kobayashi O, Sairenji M, Motohashi H. [Clinical and diagnostic significance of abdominal CT for peritoneal metastases in patients with primary gastric cancer]. Gan To Kagaku Ryoho. 2002;29(11):1925-1928. Japanese.

5) Takahashi Y. [Gastrointestinal cancer]. Gan To Kagaku Ryoho. 2004;31(8):1275-1279. Japanese.

6) Okura H. Half lives of tumor markers. Medical Technology34:753-758, 2006.

7) Ueki K, Nashimoto A, Sasaki J. A Clinicopathological study of serum ca125 concentration in gastric cancer. J Jpn Surg Assoc. 1994;55(8):1932-1937. doi: 10.3919/ringe1963.55.1932.

8) Nakata B, et al. Serum CA125 level as a predictor of peritoneal dissemination in patients with gastric carcinoma. Cancer 83:2488-2492, 1998.

9) Emoto S, et al. Clinical significance of CA125 and CA72-4 in gastric cancer with peritoneal dissemination. Gastric Cancer 15: 154-161, 2012.

10) Ginaldi S, et al. The striped colon: A new radiographic observation in metastatic serosal implants. Am J Roentgenol 134: 453-455, 1980.

11) Meyers MA, McSweeney J: Secondary neoplasms of the bowel. Radiology 105: 1-11, 1972.

12) Miyakawa K. Detection of secondary involvement of the colon from gastric carcinoma. comparison of ct and barium enema. Chiba Med J. 1994;70:245-250.

13) NCCN clinical practice guidelines in oncology: colorectal cancer screening. http://www.nccn.org

14) Pickhardt PJ, et al. Colorectal and extracolonic cancers detected at screening CT colonography in 10286 asymptomatic adults. Radiology 255: 83-88, 2010.
