# Supplementary material for: Assessment of the use of computed tomography colonography in early detection of peritoneal metastasis in patients with gastric cancer: A prospective cohort study
Source: PLoS One. 2022 Jan 25;17(1):e0261527. doi: 10.1371/journal.pone.0261527 (PMC8789127; doi:10.1371/journal.pone.0261527)
Supplement: S2 Protocol — (DOCX) [file pone.0261527.s002.docx]

臨床的に確定診断が困難な胃癌腹膜転移疑い例に対するCTコロノグラフィーのfeasibility研究

Feasibility study of CT colonography for peritoneal metastasis of gastric cancer clinically difficult to diagnose definitely

研究代表者 長　晴彦 神奈川県立がんセンター消化器外科　医長

〒241-0815横浜市旭区中尾1-1-2

TEL：045-391-5761、FAX：045-361-4692

E-mail: [choharuhiko@kcch.jp](mailto:choharuhiko@kcch.jp)

研究事務局 吉田哲雄 神奈川県立がんセンター放射線診断科　部長

〒241-0815横浜市旭区中尾1-1-2

TEL：045-391-5761、FAX：045-361-4692

E-mail: [jsbach@kcch.jp](mailto:jsbach@kcch.jp)

円谷　彰 神奈川県立がんセンター消化器外科　部長

〒241-0815横浜市旭区中尾1-1-2

TEL：045-391-5761、FAX：045-361-4692

E-mail: [tuburayaa@kcch.jp](mailto:tuburayaa@kcch.jp)

Ver1.0 2010/9/20

1. 概要
   1. シェーマ

- 1. 目的

身体所見、検査所見、画像所見から胃癌腹膜転移/再発が疑われるが確定診断に至らない症例に対し、診断能向上を目的としたCTコロノグラフィーを行う。大腸壁の腹膜播種を疑う変形の有無は担当医と放射線診断医が合同で診断し、臨床経過との相関を解析する。

- 1. 対象

1. 内視鏡生検、または手術検体において組織学的に胃癌と診断されている。
2. 身体所見、検査所見、画像所見から胃癌腹膜転移/再発が疑われる。
3. ECOG performance statusが0または1または2。
4. 経口摂取が可能。
5. 本人から文書による同意が得られている。
   1. 予定登録数と研究期間

パイロット期間の症例を含め、45例。明らかなfalse negativeを確認する目的で、15例到達時点で中間解析を施行する。研究期間は5年。

1. 目的

身体所見、検査所見、画像所見から胃癌腹膜転移/再発が疑われるが確定診断に至らない症例に対し、診断能向上を目的としたCTコロノグラフィーを行う。大腸壁の腹膜播種を疑う変形の有無は担当医と放射線診断医が合同で診断し、臨床経過との相関を解析する。

1. 背景と計画の根拠
   1. 対象
      1. 対象疾患

わが国における胃癌の死亡率は、いまだ肺癌に次いで2番目に高い。特に高度進行・再発胃癌が全体の治療成績を低下させていると思われ、SPIRITS試験での同対象に対するS-1群のMSTは13.0か月であった^１）^。中でも腹膜転移/再発は、遠隔転移を有する患者の約半数を占める、もっとも頻度が高くかつ予後不良な再発形式であり、仮に治療が行えても9カ月程度の生存しか期待できない（JCOG0106）。腹膜転移が予後不良である原因としては、細胞そのものの悪性度以外にも、以下の理由が挙げられる。

1. 腸管の狭窄や水腎症を起こしやすく、十分量の抗癌剤が投与できない
2. 画像で特定することが難しく、治療開始が遅れる
3. 腹腔内のあらゆる場所に広がる可能性があり、全体像が把握しにくい
4. 治療評価が困難なため、耐性時に速やかに次の治療に移行できない
   1. 診断
      1. 従来の画像診断

　胃癌腹膜播種の画像診断率は低い。計33の報告（US：８、EUS:5、CT:22、MRI:2、FDG-PET：5）のメタ解析^2）^では、腹膜播種の診断オッズ比はCTが66.18（95％CI: 27.28-160.53）で最も高く、EUSは13.07（6.42-26.62）、PETは12.49（2.22-70.10）、USは10.63（1.54-73.36）であった。いずれの診断方法も、診断特異度は0.96から0.99と高いが、診断感度は最も高いEUSでも0.34（0.10-0.69）、CTは0.33（0.16-0.56）という結果であり、通常胃癌の術後フォローアップで行うaxial CTでは、腹膜再発の3分の2は見逃される可能性がある。ランダム化比較試験としては韓国で行われたCT vs FDG-PETの研究^3）^がある。CTでは、腹腔内結節、腹水、腸間膜や大網の不整かつ数珠状肥厚もしくは毛羽立ち像を腹膜播種とし、PETでは腸管や腸間膜に沿ったびまん性の代謝亢進像を判断基準としている。比較の結果、CTは感度76.5％とPETの35.3％を有意に上回っていた（p=0.037）が、特異度は91.6％とPETの98.9％より劣っていた（p=0.035）。再発例だけに限った報告では、139名の胃癌患者の術後フォローアップをCTとPET/CTの両方で行った前向き研究があり、28名の再発例での診断能を報告している。結果は両者の診断能に有意差は見られなかった（感度：64.3％/53.6%、特異度：86.5％/84.7％）が、どちらか一方の方法だけで診断できた腹膜播種が18病変あったことから、両方行う方がより診断能は向上するだろうと結論付けている。診断能以外の観点では、吉川ら^4）^は腹膜転移を有する初発胃癌123例を解析し、CTの術前診断で腹膜播種陽性と診断できた正診率は37.4％と低いが、所見陽性群は陰性群と比較して有意に予後不良であったことから、予後予測因子としての意義を報告している。

- - 1. 腫瘍マーカー

　血清腫瘍マーカーとしては、胃癌患者におけるCA125値の上昇が腹膜播種の存在や広がりと相関することが知られている。高橋^5）^は、胃癌の腹膜播種における腫瘍マーカーの陽性率が、他のマーカー（CEA、CA19-9、AFP）が10％以下であるのに対し、CA125だけは42.9％と高率であり、転移臓器特異性が高いとしている。理由としては、CA125が腫瘍細胞からはほとんど産生されず、むしろ腹膜中皮細胞に存在するため、腹膜の炎症を表しているためと考えられている。そのため、感染性の腹膜炎や女性の子宮内膜症、術後早期（CA125の半減期は5～10日^6）^）などは除外しなければならない。植木ら^7）^は胃癌手術症例219例に術前にCA125を測定し、腹膜播種陽性であった47例中のCA125の感度は31.9％、特異度は93.6％であったと報告している。Nakataら^8）^はCA125の胃癌腹膜播種に対する感度を39.4％、特異度を95.7％と報告し、CTやUSなどの画像診断より優れていると報告している。Emotoら^9）^も、CA72-4など複数のマーカーを組み合わせれば感度は上がるものの、CA125単独の感度は46％であったとしている。

一方で、CA125の感度は腹膜播種の進展程度と相関するという報告が多く、腫瘍の絶対量が少ないうちは陽性とならない。Nakataらの報告ではP1（胃近傍、少量）の時のCA125の正診率は28.6％であり、CTやUSは0％であった。Emotoらの報告からは、画像で腹水がある場合の相関は高いが、P1でCA125がcutoff値を超えたのは8例中1例だけ（12.5％）だったことから、CA125に胃癌腹膜播種の画像診断を補う役割は期待できないと考えられる。

- - 1. 注腸造影

　腹膜播種が進行すると腸管狭窄をきたすことが多いため、胃癌患者の注腸造影で狭窄がみられた場合は、腹膜播種が強く疑われる。しかし、播種した腫瘍の絶対量が少ないうちの注腸造影は、”striped colon^１0）^”、”fixed transverse parallel folds^11）^”などの特徴的所見があるとされるものの、その診断感度は明らかでない。宮川^12）^は胃癌大腸転移と診断された60症例の注腸造影所見とCTとの比較を報告している。転移の診断確定の内訳は、手術：28、剖検：5、画像などの臨床判断：27であった。その結果、CTで壁の厚さが5ｍｍ以上を診断基準とした場合は80％以上の診断が得られたが、診断感度は注腸の98％（59/60）に比較し、CTでは83％（50/60）と注腸の方が優れていた。一方で、62％の症例に腹腔内腫瘤を、37％に腹水を認めたことから、多くの症例は腹膜播種由来病変であり、腹膜播種診断を目的とした場合はCTにアドバンテージがあるとしている。注腸でほとんど見落としがなかった理由の一つに、空気を注入して大腸を拡張させ、正常部と転移部の違いを際立たせたことがあげられる。仮に、CT施行時に大腸内に空気を注入した状態で撮影を行えば、CTにおける腹膜播種の感度はより向上することが期待される。

- - 1. CTコロノグラフィー

　CTコロノグラフィー（以下CTC）は1994年に初めて報告された、CT画像をコンピューターでデータ処理することで大腸の3D画像や仮想内視鏡画像を得られる画像診断法である。低侵襲で、かつ大腸内視鏡と同等の精度で大腸病変の検出が可能とされる。2011年度のNCCNガイドライン^13）^は、腫瘍性ポリープと癌を発見するスクリーニング方法として、10年ごとの大腸内視鏡検査とともに、5年ごとのCTコロノグラフィーを併記している。また、大腸以外の情報が得られることも大きな利点であり、Pickhardtら^14）^は300例に1例の割合で、大腸癌以外の癌が同定されたと報告している。しかし、大腸の壁外性の微細な変化をとらえる目的でCTCを施行した報告は、2012年10月現在、検索し得た範囲では見当たらない。

CTCの利点の一つに、診断画像の客観性・再現性が高く、標準化に適した手技であることが挙げられる。ちなみに、わが国ではCTCは2012年1月から下部消化管に対する撮影に診療報酬が適用され、16列以上のマルチスライスCTを使用し、直腸用チューブを用いて二酸化炭素を注入し、三次元画像処理を行った場合に600点の加算が算定できる。

- 1. 研究デザイン
     1. エンドポイント

Primary endpointは診断感度。従来法の診断感度は報告により幅があるが、現在主流のMDCTでの感度はおよそ60％と想定される。CTCでの感度は期待値75％、閾値55％と設定するとSimon 2-stage designでα=0.05, β＝0.2で43例必要になる．再発の確定診断は、ほとんどの症例で組織学的な確認ができないため困難であるが、CTC施行後、大量腹水/消化管狭窄/水腎症など臨床的に明らかに腹膜播種再発に至ったものとする。期間は化学療法施行中の症例で6か月、非施行例で3か月以内とする。CTC所見陰性で、同期間内に明らかに腹膜再発した場合はfalse negativeとし、15例の段階で中間解析を行い、15例中6例false negativeが判明した場合は登録を中断する。

- - 1. 患者集積見込み

　　神奈川県立がんセンターでの、胃癌術後の全症例に対する再発率は、約10-20％である。年間症例数では約30例程度と思われる。その中で、腹膜播種再発のみの疑いが生じる症例は、5例程度と予想される。切除の対象とならない進行例や、他施設での症例を含めても、年間症例数は8例程度と思われる。上記統計結果や同意取得を考慮して、登録予定数を5年で45例とした。

1. 患者選択基準

想定される対象患者は、大腸に変形をきたす程度の播種であることから、胃癌取扱い規約第12版でのP3相当のものが大部分であると思われる。ただし、本研究では、早期胃癌の術後など、臨床上・統計学上播種の可能性は低くても、下記画像所見を満たす場合は、播種陰性であることを確認する目的で対象に含めることを許容する。

- 1. 適格基準
  2. 内視鏡生検、または手術検体において組織学的に胃癌と診断されている。
  3. 身体所見（腹部膨満、腹痛、テネスムス、悪心嘔吐）、検査所見（腫瘍マーカーの正常からの逸脱）、画像所見から胃癌腹膜転移/再発が疑われる（腹水、腸管壁肥厚、腹膜脂肪組織濃度の不整な上昇、腹腔内結節）。
  4. ECOG performance statusが0または1または2。
  5. 経口摂取が可能。
  6. 本人から文書による同意が得られている。
  7. 除外基準

1. 消化管の前処置ができない。
2. 明らかな消化管通過障害を有する。
3. 大量腹水。
4. 直腸からの送気ができない。
5. 登録
   1. 登録の手順

適格・除外基準を確認し、登録表を下記データセンターにFAX送信する。

データセンター：　高橋利香

神奈川県立がんセンター消化器外科

TEL：045-391-5761（内線2633）

FAX：045-361-4692

1. 検査
   1. 標準検査手順
      1. 前処置

ブラウン変法を採用。検査前日の食事は低残渣食とし、下剤はクエン酸マグネシウム（マグコロールP）50ｇおよびピコスルファートナトリウム（ラキソベロン）1本を検査前日15時に服用。検査当日朝にレシカルボン坐薬1個を挿肛。

- - 1. 撮影
  1. 抗コリン剤（ブチルスコポラミン）1Aまたはグルカゴン1Aを筋注（心疾患、緑内障、前立腺肥大、糖尿病などがある場合は原則不要）
  2. 左側臥位とし、経肛門的にバルーンカテーテルを留置し、CO2自動注入器を用いて初めは低圧から開始し、徐々に圧を上げ、深部大腸にCO2を送る。総送気量は800～2000mLが目安だが個人差もあり、自動注入器の表示圧を確認しながら行う。圧の変化が少なくなれば、腸管が十分に拡張したと考えてよい。
  3. 苦痛の程度を聞き、軽度の腹部膨満感を訴えたところで注入圧を保つようにして、仰臥位で撮影し、結腸の拡張を確認。拡張が十分でない場合は送気を追加し再度仰臥位で撮影。十分な拡張が得られている場合は、必要に応じて側臥位、腹臥位の撮影を追加する。CTは16列以上のMDCTでおこなう。
  4. 画像処理と腹膜播種診断

　得られた画像を、1mmの再構成で三次元画像処理ワークステーションに転送し、大腸3D画像と仮想内視鏡画像を作成する。放射線診断医は、大腸壁の異常な変形およびその他の臓器に関する読影レポートを作成。最終的な腹膜播種の診断は、読影レポートを基に主治医が判断する。

1. 予期される有害事象

　CTC施行に伴い、消化管穿孔、消化管出血、肛門出血などが起こりうるため、有害事象が生じた場合には適切に対処する。

1. 倫理的事項

本試験に関係する全ての研究者は、ヘルシンキ宣言（日本医師会http://www.med.or.jp/wma/）および臨床研究に関する倫理指針（厚生労働省告示第255号：http://www.mhlw.go.jp/topics/2003/07/tp0730-2.html）　に従って本試験を実施する。

- 1. 患者への説明および同意の取得

担当医師は，患者を登録する前に，患者に対し施設の倫理審査委員会（またはInstitutional Review Board，IRB）で承認の得られた説明文書を渡し，口頭で十分に説明し，本人の自由意思による同意を同意書で得るものとする。同意書には，説明を行った担当医師のほか，研究協力者が補足的な説明を行った場合には，当該研究協力者が，各々その日付を記入の上で記名捺印または署名し，対象者は同意日を記入の上で記名捺印または署名する。同意書の写しは対象者に提供し，原本は施設で保存する。

- 1. プライバシーの保護と患者識別

研究実施に係わる生データ類および同意書等を取扱う際は，対象者の秘密保護に十分配慮する。また，病院外に提出する症例報告書の作成，取扱い等においても，登録割付時に発行される「症例登録番号」と施設内で設定する「対象者識別コード」により対象者を特定し，その秘密保護について配慮する。本研究で得られた対象者のデータは本研究の目的以外には使用しない（本研究以外の目的で使用する場合は，必要に応じ別途同意を対象者から取得する）。なお，研究の結果を公表する際も対象者を特定できる情報は使用しない。

- 1. 施設の倫理審査委員会（Institutional Review Board，IRB）の承認

本研究の開始に先立ち，各施設は必要な書類を提出し，施設の倫理審査委員会（IRB）の迅速審査等で本研究への参加を承認されなければならない。

- 1. 利益相反（conflict of interest）

本研究の計画，実施，発表に影響する可能性のある利益相反（conflict of interest）は想定していない。利益相反（conflict of interest）とは，研究成果に影響するような利害関係を指し，金銭および個人の関係を含む。

1. 研究組織
   1. 研究代表

長　晴彦

神奈川県立がんセンター　消化器外科

〒241-0815　神奈川県横浜市旭区中尾1-1-2

TEL: 045-391-5761　FAX: 045-361-4692　E-mail: choharuhiko@kcch.jp

- 1. 研究事務局

吉田哲雄 神奈川県立がんセンター放射線診断科　部長

〒241-0815横浜市旭区中尾1-1-2

TEL：045-391-5761、FAX：045-361-4692

E-mail: [jsbach@kcch.jp](mailto:jsbach@kcch.jp)

円谷　彰 神奈川県立がんセンター消化器外科　部長

〒241-0815横浜市旭区中尾1-1-2

TEL：045-391-5761、FAX：045-361-4692

E-mail: [tuburayaa@kcch.jp](mailto:tuburayaa@kcch.jp)

- 1. データセンター

高橋利香

神奈川県立がんセンター消化器外科

TEL：045-391-5761（内線2633）

FAX：045-361-4692

- 1. 研究計画書作成委員

長　晴彦　神奈川県立がんセンター消化器外科

吉川貴己　神奈川県立がんセンター消化器外科

吉田哲雄　神奈川県立がんセンター放射線診断科

島田英昭　東邦大学外科学講座　一般・消化器外科

1. 参考文献
   - 1. Koizumi W, et al. S-1 plus cisplatin versus S-1 alone for first-line treatment of advanced gastric cancer (SPIRITS trial): a phase III trial. Lancet Oncol 9: 215-221, 2008.
     2. Wang Z, Chen JQ. Imaging in assessing hepatic and peritoneal metastases of gastric cancer: a systematic review. BMC Gastroenterol 11: 19, 2011.
     3. Kim DW, Park SA, Kim CG. Detecting the recurrence of gastric cancer after curative resection: comparison of FDGPET/CT and contrast-enhanced abdominal CT. J Korean Med Sci 26: 875-880, 2011.
     4. 吉川貴己、他.　CTによる胃癌腹膜転移診断の有用性。癌と化学療法29：1925-1928、2002.
     5. 高橋豊、他.　消化管腫瘍。癌と化学療法31：1275-1279、2004.
     6. 大倉久直.　腫瘍マーカーの半減期。Medical Technology34:753-758, 2006.
     7. 植木匡、他.　進行胃癌における血清CA125の臨床病理学的検討。日臨外医会誌55：1932-1937、1994.
     8. Nakata B, et al. Serum CA125 level as a predictor of peritoneal dissemination in patients with gastric carcinoma. Cancer 83:2488-2492, 1998.
     9. Emoto S, et al. Clinical significance of CA125 and CA72-4 in gastric cancer with peritoneal dissemination. Gastric Cancer 15: 154-161, 2012.
     10. Ginaldi S, et al. The striped colon: A new radiographic observation in metastatic serosal implants. Am J Roentgenol 134: 453-455, 1980.
     11. Meyers MA, McSweeney J: Secondary neoplasms of the bowel. Radiology 105: 1-11, 1972.
     12. 宮川国久.　胃癌の大腸転移の画像診断。千葉医学70：245-250、1994.
     13. NCCN clinical practice guidelines in oncology: colorectal cancer screening. <http://www.nccn.org>
     14. Pickhardt PJ, et al. Colorectal and extracolonic cancers detected at screening CT colonography in 10286 asymptomatic adults. Radiology 255: 83-88, 2010.
